# Real-world outcomes of hybrid obesity care using digital coaching and GLP-1 therapy in a multi-ethnic Asian setting

**DOI:** 10.1038/s41366-026-02062-x

**Published:** 2026-04-09

**Authors:** Shahmir H. Ali, Michelle H. Lee, Kyle Xin Quan Tan, Chu Jing Wong, Faidon Magkos, Ian Yi Han Ang, Sue-Anne Toh

**Affiliations:** 1 Saw Swee Hock School of Public Health, National University of Singapore and National University Health System, Singapore, Singapore; 2 Department of Medicine, Yong Loo Lin School of Medicine, National University of Singapore and National University Health System, Singapore, Singapore; 3 NOVI Health, Singapore, Singapore; 4 Department of Nutrition, Exercise and Sports, University of Copenhagen, Copenhagen, Denmark; 5 Department of Medicine, National University Hospital, National University Health System, Singapore, Singapore

**Keywords:** Weight management, Patient education

## Abstract

**Background:**

Obesity remains a major health challenge globally and in Asia, driving cardio-metabolic disease risks. Glucagon-like peptide-1 receptor agonists (GLP-1 RAs) and mobile health (mHealth) coaching each demonstrate weight loss efficacy, but real-world evidence for hybrid models combining these treatments remains limited, especially in multi-ethnic Asian settings.

**Methods:**

We evaluated real-world outcomes among 708 adults enrolled in NOVI Optimum Plus, a physician-led obesity program in Singapore that integrates GLP-1 RA pharmacotherapy with app-based lifestyle coaching. Data on weight, metabolic indicators, and engagement were extracted from clinical records and the mHealth platform. Linear mixed models estimated changes over 6–18 months, stratified by engagement, metabolic status, and ethnicity.

**Results:**

Participants (mean age 42.1 years; 64.1% female) were primarily East Asian (45.5%), European (26.8%), South Asian (13.3%), and Southeast Asian (10.3%). Most received semaglutide (86% oral 14 mg). At 12 months, mean weight loss was 12.7% (95% CI: –14.0, –11.3) and BMI dropped by 4.1 points, with further weight loss reaching 14.7% at 18 months. Systolic blood pressure decreased by 11.5 mmHg, body fat percentage by 8.8%, waist-to-hip ratio improved from 0.83 to 0.80, and HbA1c declined by 0.6%. Greater app engagement was linked to 2.0–2.2% additional weight loss, 0.72 kg/m² more BMI reduction, and up to 2.9 mmHg greater systolic BP drop. More frequent health coach contact contributed modest added improvements for weight and BMI. Weight loss was significantly lower among East Asians and those with hyperglycemia.

**Conclusion:**

In this real-world Asian setting, hybrid obesity care combining GLP-1 RAs with digital coaching produced clinically meaningful, sustained weight and metabolic improvements. Higher engagement consistently enhanced outcomes, supporting scalable integrated models tailored for diverse populations.

## Introduction

Obesity, a complex chronic condition caused by a range of biological, behavioral, and environmental factors, poses a major global public health challenge [1], affecting nearly 890 million people worldwide [2] and significantly contributing to cardio-renal-metabolic disease morbidity and mortality [3]. In Singapore, where obesity prevalence is substantial [4] and cardiovascular mortality is high [5], addressing this condition is a recognized priority, underscored by recent WHO calls for intensified regional action [6].

While lifestyle interventions are the cornerstone of obesity management, self-directed efforts often fall short [7]. Structured support is crucial, and hybrid care models integrating digital health technologies (mHealth) with human medical care and coaching have emerged as effective and scalable approaches to enhance personalized lifestyle modification [8–10]. Concurrently, pharmacological treatment has advanced significantly with the advent of GLP-1 receptor agonists (GLP-1 RAs), such as liraglutide, semaglutide and tirzepatide, which demonstrate substantial efficacy for weight loss [11–14] and are approved for obesity management [15].

Despite the individual promise of both technology-enhanced lifestyle coaching and GLP-1 RA pharmacotherapy, there remains a critical gap in understanding their combined impact within integrated care models. Here, we provide evidence on the real-world effectiveness of a physician-led, hybrid digital–pharmacological obesity care model combining these two treatment modalities, in a diverse multi-ethnic patient population from Singapore.

## Methods

### Study design, site and patients

This analysis used real-world data from patients attending a metabolic health clinic (NOVI Health) in Singapore, a city-state in tropical Southeast Asia with a population of 6.04 million [16]. The patients selected were those who had enrolled in NOVI’s Optimum Plus, an mHealth counseling and GLP-1 RA-based weight care intervention program lasting at least 3 months, throughout the period of its inception from March 30, 2022 to June 30, 2024. The program was delivered within a private healthcare setting in Singapore and was largely self-paid by participants, with limited insurance subsidies in some cases, reflecting the predominantly out of pocket financing model for private metabolic care in the Singapore context. Participants were recruited through multiple channels, including physician referrals, online content marketing on social media and the clinic’s website, direct searches by individuals seeking treatment, and referrals from existing patients. These platforms and referral pathways were accessible to both residents and non-residents living in Singapore. To be eligible for the program, patients were required to either [1] have obesity (BMI ≥ 27.5 kg/m² for Asians or ≥30 kg/m² for non-Asians), or [2] be overweight (BMI ≥ 23 kg/m² for Asians or ≥25 kg/m² for non-Asians) with at least one weight-related comorbidity. While some patients who did not meet these criteria were allowed to enroll for individualized valid clinical reasons (e.g., lower BMI with specific health concerns related to excess adiposity), this analysis focuses only on patients who met the formal program eligibility. Additional inclusion criteria were age of at least 21 years, enrollment in the intervention for at least 3 months, and having at least one weight measurement taken ≥3 months after baseline. Among 1753 patients enrolled in NOVI Optimum Plus, 1055 remained in the program for at least 3 months (60.2%), of whom 727 had sufficient weight data. After restricting to those meeting formal eligibility criteria, the final sample for analysis comprised 708 participants. Because this was a retrospective study using all eligible patients within a defined program period, no a priori power calculation was performed. However, the resulting analytic sample was sizeable and comparable to or larger than those used in prior real-world evaluations of GLP-1 RA-based weight loss programs [17, 18]. All data were extracted from the electronic medical records and the mobile health app database at NOVI Health and patient consent was waived given its retrospective nature and use of only deidentified data. This study was approved by the Institutional Review Board of the National University of Singapore (NUS-IRB-2023-805).

### Intervention

**Fig. 1:**
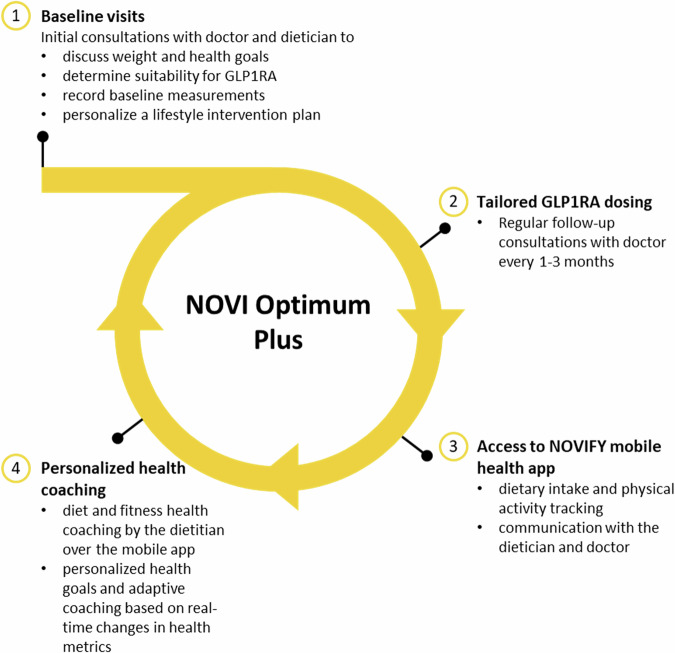
Overview of the NOVI Optimum Plus weight loss programme.

The NOVI Optimum Plus is a medical weight loss program where physician-directed GLP-1 RA therapy is coupled with personalized diet and physical activity counseling by a dedicated care team, and continuous support through a proprietary mobile health app, NOVI Health. The app’s key functions include tracking of dietary intake and physical activity levels, asynchronous messaging with the doctor and dietician, and provision of educational content tailored for each patient. The program is standardized to include the following components (Fig. 1): (1) initial consultations with a doctor and health coach (dietitian and/or fitness coach) to determine weight loss and health goals, assess suitability for GLP-1 RA, record baseline measurements, and personalize a lifestyle intervention plan; (2) tailored GLP-1 RA dosing and regular follow-up consultations with the doctor every 1–3 months; (3) access to the mobile app that allows dietary intake and physical activity tracking and communication via text messaging with the health coach and doctor; and (4) personalized diet and physical activity coaching provided by the health coach over the mobile app in response to the real-time changes in the patient’s health metrics (Supplementary File 1). In this cohort, GLP-1 RAs prescribed included liraglutide and semaglutide, and less commonly, dulaglutide. Dosage and mode of administration (oral or subcutaneous) depended on the patient’s clinical condition(s), receptiveness and response to the drug, as well as availability in the local market (Table 1).

**Table 1:** Baseline characteristics of program patients analyzed (*n* = 708).

Variable	
Age, mean (SD), *n* = 708	42.11 (9.10)
Sex, *n* (%), *n* = 708	
Female	454 (64.1)
Male	254 (35.9)
Nationality, *n* (%), *n* = 699	
Non-Singaporean	340 (48.6)
Singaporean	359 (51.4)
Ethnicity (region of origin), *n* (%), *n* = 708	
East Asian	322 (45.5)
South Asian	94 (13.3)
Southeast Asian	73 (10.3)
European	190 (26.8)
Other/Mixed	29 (4.1)
BMI, mean (SD), *n* = 708	32.2 (5.7)
WHR, mean (SD), *n* = 397	0.93 (0.07)
SBP, mean (SD), *n* = 394	131.5 (16.1)
DBP, mean (SD), *n* = 394	88.4 (10.1)
Fat %, mean (SD), *n* = 42	38.4 (8.7)
Cholesterol, mean (SD), *n* = 74	200.9 (41.6)
HDL Cholesterol, mean (SD)	54.1 (13.7)
LDL Cholesterol, mean (SD)	120.7 (36.0)
Triglycerides, mean (SD), *n* = 88	142.8 (76.7)
HbA1c, mean (SD), *n* = 118	5.9 (0.9)
Total health conditions^a^, mean (SD), *n* = 708	1.72 (1.09)
Obesity, *n* (%), *n* = 708	637 (90.0)
Dyslipidemia, *n* (%), *n* = 708	180 (25.4)
Hypertension, *n* (%), *n* = 708	129 (18.2)
Hyperglycemia, *n* (%), *n* = 708	99 (14.0)
Program length [days], (median, IQR), *n* = 708	240 [180, 360]
Monthly (median, IQR)	
Doctor consults, *n* = 708	0.57 [0.42, 0.88]
Health coach consults, *n* = 708	0.11 [0.00, 0.17]
Logs on app, *n* = 563	34.7 [13.3, 78.8]
Messages on app, *n* = 563	3.91 [1.33, 7.80]
Time [min] on app, *n* = 563	19.0 [3.7, 54.6]
Care delivery modality	
Fully tele-visits	244 (34.5)
Hybrid	269 (38.0)
Fully in person	195 (27.5)
Type of GLP1-RA medication, *n* (%), *n* = 708	
Dulaglutide, 0.75 mg, subcutaneous	1 (0.1)
Dulaglutide, 1.5 mg, subcutaneous	10 (1.4)
Liraglutide, 3 mg, subcutaneous	2 (0.3)
Semaglutide, 0.5 mg, subcutaneous	191 (27.0)
Semaglutide, 1 mg, subcutaneous	249 (35.2)
Semaglutide, 3 mg, oral	369 (52.1)
Semaglutide, 7 mg, oral	525 (74.2)
Semaglutide, 14 mg, oral	608 (85.9)

*WHR* waist-to-hip ratio, *SBP* systolic blood pressure, *DBP* diastolic blood pressure.

^a^Conditions were identified using ICD 10 diagnostic groupings: obesity (E66 and related abnormal weight codes), dyslipidemia (E78), hypertension (I10–I15 and elevated blood pressure codes), and hyperglycemia (E09–E11 and O24).

### Data collection and outcome measures

Patients’ anthropometric (i.e. height, weight, and waist and hip circumferences) and blood pressure measurements were taken by a nurse or trained staff. For patients who were seen remotely via tele-consultation, height and weight were self-reported; however, available in-clinic measurements of height and weight were also extracted to inform subsequent sensitivity analyses. Depending on the patient’s medical needs other than weight loss, body fat % was measured using a bio-impedance scale (model H01A, GMM Technoworld Pte Ltd, Singapore), and metabolic biomarkers such as HOMA-IR, HbA1c, cholesterol, and triglyceride concentrations were determined at an accredited clinical laboratory (Innoquest Diagnostics, Singapore). Indices of engagement included the total number of formal doctor and health coach consultations, as well as NOVI Health mobile app usage (i.e. total logs, number of messages, and total time spent). Total logs are logs keyed in manually by users included meals, activities, glucose, insulin, weight and blood pressure values. Number of messages is the total number of messages sent by a patient on the mobile app and total time spent is the amount of active time a patient spent on the mobile app between the first and last visit. The primary outcome was the change in body weight. Secondary outcomes were the changes in other cardiometabolic measures. Additionally, health conditions were categorized, informed by the ICD codes provided for each condition. Similarly, for patients without available data on ethnicity, a probable ethnicity was deduced by triangulating name, nationality, and other notes recorded during program visits.

### Statistical analysis

Descriptive analyses of participant socio-demographic, baseline health, and engagement indicators were first conducted. Descriptive longitudinal analyses for percentage weight change and BMI were performed using LOESS-smoothed curves to examine average trajectories over time. Statistical analyses were conducted through a series of linear mixed models (accounting for participant-level random effects) to assess changes in outcomes across time, adjusting for age, sex, nationality, ethnicity, and total number of health conditions, as well as the interaction effects of sex and total number of health conditions—the variables selected were informed by bivariable analyses of the data and past evidence to suggest potential confounding or effect modification [19]. Interaction analyses were conducted to evaluate whether changes in percentage weight differed between participants with and without hyperglycemia, and whether variations were observed across ethnic groups. Outlier data points (those above or below 3 standard deviations) were identified and removed prior to these statistical analyses. A sensitivity analysis was also conducted using the subset of BMI and weight change datapoints collected through in-clinic measurements only. Given the non-linear relationship between months since starting the intervention and all outcomes observed in descriptive analyses and supported by similar weight loss interventions [20, 21], the time variable (months) was log-transformed prior to analyses. Constructed linear mixed models were then used to generate estimates (including 95% confidence intervals, CIs) of each analyzed outcome at baseline, 6 months, 12 months, and 18 months.

Subsequently, among all participants in the final analytical sample, predictors of greater engagement were evaluated through a series of logistic regression models to assess the association between age, sex, nationality, ethnicity, and prevalent health conditions with higher or above median program length, monthly doctor and health coach consults, and monthly total app logs, app time, and messages. Finally, another series of linear mixed models were constructed to explore differential changes in health outcomes across different levels of engagement (monthly total logs, app time, messages, doctor consults, and health coach consults) by adjusting the earlier linear mixed models to include an interaction term for each variable. Differences across quartiles were evaluated across all engagement variables. For health coach engagement data, a median split was used instead of quartile due to the distribution of the data. Visualizations were also made to describe the modeled monthly estimates of key outcomes at different levels of engagement. All analyses were conducted in R (version 4.3.0).

## Results

### Participants

Table 1 presents the baseline characteristics of the 708 participants included in the analysis. The majority were female (64.1%), with a mean age of 42.1 years (SD = 9.1). The cohort was ethnically diverse, consisting primarily of East Asians (45.5%), followed by Europeans (26.8%), South Asians (13.3%), and Southeast Asians (10.3%). Common comorbidities included dyslipidemia (25.4%), hypertension (18.2%), and hyperglycemia (14.0%). At baseline, participants were prescribed a range of GLP-1 RA, with semaglutide being the most commonly prescribed agent across both injectable and oral formulations (Table 1). Aside from GLP-1 RA therapies, medication use data (not displayed) were limited to prescriptions issued within the NOVI Health system and may not fully capture medications obtained outside of the program. Within this context, 4.4% of participants were prescribed statins, 2.6% were prescribed antihypertensive medications, and 14.5% received metformin or combination glucose-lowering therapies, figures that likely underestimate true medication use given the substantially higher prevalence of underlying cardiometabolic conditions in the cohort.

### Clinical health outcomes

**Fig. 2:**
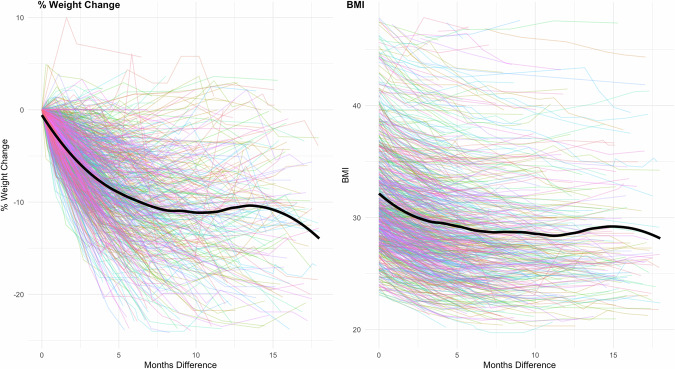
Percentage weight change and BMI of NOVI Optimum Plus program participants at different stages of the program^a^. ^a^Black line represents a LOESS-smoothed curve, providing a trend of the average trajectory over time.

At baseline, participants had an average BMI of 32.2 kg/m^2^ (SD = 5.7) and a mean waist-to-hip ratio of 0.93 (SD = 0.07) (Table 1). Other metabolic indicators included an average systolic blood pressure of 131.5 mmHg (SD = 16.1), body fat % of 38.4 (SD = 8.7), and HbA1c of 5.9% (SD = 0.9). As shown in Fig. 2, unadjusted weight and BMI trends exhibited a steady decline over time, with the LOESS-smoothed curve highlighting an initial sharp drop, followed by a gradual plateau. Weight loss was most rapid in the first six months, with continued but slower reductions thereafter. A similar pattern was observed for BMI, with a steady decrease across the follow-up period.

Table 2 presents adjusted estimates of clinical outcomes at key time points, which were all statistically significant, except for triglycerides and total, HDL, and LDL cholesterol concentrations (Supplemental File 3). By 6 months, participants experienced an average weight reduction of 9.2% (95% CI: −10.4, −7.8), which increased to 12.7% (95% CI: −14.0, −11.3) at 12 months and 14.7% (95% CI: −16.0, −13.3) at 18 months. Interaction analyses (not displayed) revealed significant differences in weight change trajectories by hyperglycemia status and ethnicity. Participants with hyperglycemia experienced a significantly attenuated reduction in weight over each log-month of follow-up compared to those without hyperglycemia (interaction estimate = +1.15, 95% CI: 0.74–1.55; *p* < 0.001). Ethnic differences were also observed: compared to East Asian participants, those of European (–0.59, 95% CI: –0.87 to –0.31; p < 0.001) and Other/Mixed (–0.97, 95% CI: –1.59 to –0.35; *p* = 0.002) backgrounds experienced significantly more weight reduction per log-month.

**Table 2:** Adjusted^a^ estimates of health outcomes at different lengths of NOVI Optimus Plus program^b^.

	Month 0	Month 6	Month 12	Month 18
Variable	Estimate (95%CI)	Estimate (95%CI)	Estimate (95%CI)	Estimate (95%CI)
% Weight change		−9.2 (−10.4, −7.8)	−12.7 (−14.0, −11.3)	−14.7 (−16.0, −13.3)
BMI	31.9 (29.9, 33.9)	28.9 (27.0, 30.9)	27.8 (25.8, 29.8)	27.1 (25.2, 29.1)
WHR	0.83 (0.80, 0.86)	0.81 (0.78, 0.84)	0.80 (0.78, 0.83)	0.80 (0.77, 0.83)
SBP^c^	108.4 (102.6, 114.2)	100.1 (94.4, 105.8)	96.9 (91.0, 102.8)	95.0 (89.1, 101)
Body fat %	38.6 (32.6, 44.5)	32.2 (26.7, 37.8)	29.8 (24.2, 35.4)	28.4 (22.6, 34.1)
Triglycerides	114.8 (70.9, 158.6)	99.2 (57.6, 140.8)	93.2 (49.4, 137.0)	89.7 (43.9, 135.4)
HbA1c	5.1 (4.8, 5.4)	4.7 (4.3, 5)	4.5 (4.2, 4.9)	4.4 (4.1, 4.8)

*WHR* waist-to-hip ratio, *SBP* systolic blood pressure.

^a^Adjusting for age, sex, nationality, ethnicity, and total health conditions, and interaction effects of sex and total health conditions. Datapoints analyzed for each outcome: % Weight Change (3735, 37 outliers removed), BMI (3697, 44 outliers removed), WHR (1922, 7 outliers removed), SBP (1918, 12 outliers removed), Fat % (357, 0 outliers removed), Total Cholesterol (386, 2 outliers removed), HbA1c (473, 5 outliers removed), Triglycerides (412, 5 outliers removed).

^b^Changes in Total, HDL, and LDL Cholesterol were not significant (Supplemental File 3), thus estimates not displayed.

^c^Only SBP findings are displayed, as DBP findings were similar.

Similarly, BMI decreased from 31.9 kg/m^2^ (95% CI: 29.9, 33.9) at baseline to 28.9 kg/m^2^ (95% CI: 27.0, 30.9) at 6 months, 27.8 kg/m^2^ (95% CI: 25.8, 29.8) at 12 months, and 27.1 kg/m^2^ (95% CI: 25.2, 29.1) at 18 months. Coefficients for BMI change and percentage weight reduction remained largely consistent in sensitivity analyses limited to datapoints from in-clinic visits (Supplemental File 3). Metabolic indicators also improved significantly from baseline to 18 months of follow-up: waist-to-hip ratio declined from 0.83 (95% CI: 0.80, 0.86) to 0.80 (95% CI: 0.77, 0.83); systolic blood pressure decreased from 108.4 mmHg (95% CI: 102.6, 114.2) to 95.0 mmHg (95% CI: 89.1, 101.0); body fat % decreased from 38.6% (95% CI: 32.6, 44.5) to 28.4% (95% CI: 22.6, 34.1); and HbA1c declined from 5.1% (95% CI: 4.8, 5.4) to 4.4% (95% CI: 4.1, 4.8).

### Engagement with program

Program engagement indicators, summarized in Table 1, varied widely. The median program duration was 240 days (IQR: 180–360 days). Participants engaged in a median of 0.57 (IQR: 0.42–0.88) doctor consults per month, while health coach consults were less frequent, with a median of 0.11 (IQR: 0.00–0.17) per month, consistent with the program structure in which physician consultations were required for GLP-1 RA prescribing, whereas engagement with health coaching was highly encouraged but could not be mandated. Furthermore, health coaching for behavioral and lifestyle support could be delivered via asynchronous messaging via the app, which could have reduced the need for health coach consults. Digital engagement levels were substantial, with participants logging a median of 34.7 (IQR: 13.3–78.8) entries per month, sending 3.9 (IQR: 1.3–7.8) messages per month, and spending a median of 19.0 (IQR: 3.7–54.6) minutes per month on the app. Supplemental File 2 shows that program engagement varied significantly by ethnicity, nationality, sex, and health conditions. Compared to East Asians, South Asians (average OR = 0.56, 95% CI: 0.33, 0.94) and Southeast Asians (OR = 0.38, 95% CI: 0.20, 0.70) had lower odds of remaining in the program for more than 9 months. Singaporeans were more likely than non-Singaporeans to engage via monthly messages (OR = 2.07, 95% CI: 1.32, 3.29) and app use (OR = 2.17, 95% CI: 1.37, 3.47). Male participants had lower odds of receiving monthly health coach consults (OR = 0.61, 95% CI: 0.44, 0.85) compared to females. Participants with hyperglycemia had higher odds of long-term retention (OR = 2.16, 95% CI: 1.32, 3.56) but were less likely to engage via messages (OR = 0.50, 95% CI: 0.31, 0.82) compared to those without hyperglycemia. By contrast, patients with dyslipidemia had lower odds of long-term retention (OR = 0.56, 95% CI: 0.37, 0.82) but were more likely to engage via messages (OR = 1.69, 95% CI: 1.16, 2.48) compared to those without dyslipidemia.

**Fig. 3:**
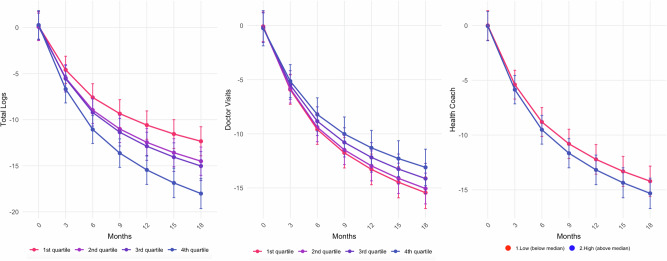
Longitudinal, adjusted^a^ predicted percentage weight change of NOVI Optimus Plus participants by varying degrees of monthly total logs on NOVI Health app, doctor visits, and health coach consultations^b^. ^a^Adjusting for age, sex, nationality, ethnicity, total health conditions, and interaction effects of sex and total health conditions. ^b^Due to the distribution of health coach engagement data, a median split was used instead of quartile.

Interaction analyses (Supplemental File 3) evaluated the relationship between engagement and changes in weight, BMI, waist-to-hip ratio, systolic blood pressure, and HbA1c levels. Figure 3 specifically illustrates these associations for weight change. Greater digital engagement (measured by total logs, messages sent, and app usage time) was associated with greater reductions in weight, BMI, waist-to-hip ratio, and systolic blood pressure. Participants in the highest quartile of total logs per month experienced 2.0% (95% CI: −2.4, −1.6) greater weight loss, 0.65 kg/m^2^ (95% CI: −0.79, −0.52) greater BMI reduction, and 2.9 mmHg (95% CI: −5.0, −0.9) greater reduction in systolic blood pressure, compared to those in the lowest quartile. Higher app usage was linked to a 2.2% (95% CI: −2.5, −1.8) greater weight loss, 0.72 kg/m^2^ (95% CI: −0.85, −0.58) greater BMI reduction, and 2.4 mmHg (95% CI: −4.53, −0.24) greater systolic blood pressure reduction. More frequent health coach consults (above the median) were associated with 0.4% (95% CI: −0.6, −0.1) greater weight loss, and 0.13 kg/m^2^ (95% CI: −0.22, −0.05) greater BMI reduction.

## Discussion

In this real-world evaluation of a hybrid weight loss intervention, we found that combining physician-led GLP-1 RA pharmacotherapy with app-based digital health coaching was associated with meaningful improvements in body weight, body composition and cardiometabolic health among patients at risk. Participants experienced sustained reductions in BMI, waist-to-hip ratio, systolic blood pressure, body fat %, and HbA1c, indicating that the weight loss achieved translated into broader clinical benefits. Higher engagement with the care team and program, facilitated by the app’s tools for self-logging and messaging, was consistently associated with better outcomes, suggesting that digitally delivered behavioral support by the health coach and physician plays an important role in optimizing response to pharmacotherapy. These findings demonstrate the feasibility and value of delivering integrated obesity care through hybrid digital models in real-world Asian healthcare settings, where such approaches remain relatively underexplored.

A key finding from this study was the substantial and sustained weight loss achieved over the 18-month follow-up period. The average reductions closely align with those reported in major randomized controlled trials of GLP-1 RAs, including one evaluating once-weekly semaglutide, which reported a 14.9% mean weight loss at 68 weeks, and another assessing daily liraglutide, which found an average 8.0% reduction at 56 weeks [11, 12]. Improvements among our patients are comparable to, and in some cases greater than, results from other real-world settings, reinforcing that the ~12–15% losses observed here represent a clinically meaningful effect consistent with established GLP-1 RA performance. For example, a Canadian cohort study reported a 12.2% total body weight loss after 17 months of GLP-1 RA therapy, while a U.S. primary care study documented a more modest reduction, with an average BMI decrease of 0.83 kg/m^2^ over 12 months [22, 23]. Recent large-scale real-world data report more modest 1-year weight loss of ~9%, largely attributed to high discontinuation rates and predominately low maintenance doses [24]. The slightly greater improvements in our study compared to past studies is likely explained by several program features: patients could titrate or switch between GLP-1 RAs based on clinical response, most participants did not have diabetes (a group that tends to experience greater weight change with GLP-1 RAs) [25], and the behavioral coaching delivered in this hybrid model is more intensive than the lighter-touch educational or lifestyle support typically offered in clinical or real-world studies, which may enhance medication effects. These observations suggest that, when delivered with clinical oversight and behavioral support, GLP-1 RA-based interventions can produce real-world outcomes on par with those observed in clinical trials. They also highlight the potential of hybrid care models that combine pharmacotherapy with digital tools to support long-term weight management across diverse settings.

Beyond weight loss, participants also experienced significant improvements in key metabolic parameters, including systolic blood pressure, waist-to-hip ratio, body fat %, and HbA1c. These changes reinforce that the weight loss achieved had meaningful physiological effects, translating into reductions in established cardiometabolic risk factors. Such improvements are important given the strong links between obesity, hypertension, insulin resistance, and cardiovascular disease [26]. The consistency of these effects across multiple domains suggests that integrated pharmacological and behavioral interventions may offer a comprehensive strategy for improving metabolic health, not just body weight.

Participants who engaged more actively with the program through self-logging, time spent in the app, and messaging with health coaches experienced greater improvements in both weight loss and cardiometabolic outcomes. This highlights the value of combining pharmacological treatment with behavioral support delivered conveniently through digital platforms. While an earlier meta-analysis found no consistent added benefit of combining health coaching with smartphone apps compared to apps alone [27], more recent evidence suggests that human coaching can significantly enhance the impact of digital interventions. A 2024 randomized trial found that adding coaching to an adaptive wireless feedback system led to greater weight loss than using the system alone, with improvements mediated by increased self-monitoring, motivation, and self-efficacy [28]. In our study, engagement was higher among women, individuals without hyperglycemia, and those of Southeast Asian ethnicity, suggesting that these groups may be more inclined to utilize and benefit from coaching features. However, further research is needed to better understand what drives or limits engagement across different subgroups and to inform strategies for more tailored and equitable implementation.

This study has several strengths. It provides one of the first real-world evaluations of a GLP-1 RA-based weight loss program integrated with physician-led and app-enabled behavioral health coaching support in an Asian context, with a multi-ethnic population. The use of routinely collected clinical and engagement data enabled the examination of long-term outcomes across multiple metabolic indicators, while granular app usage data allowed for analysis of engagement–outcome relationships. However, several limitations should be acknowledged. As a single-site analysis of a self-enrolled commercial cohort, the population may not fully represent the broader diversity of individuals with obesity, particularly those with limited access to digital tools or private care settings. Moreover, health conditions were systematically documented during physician led clinical assessments within the program, this analysis was conducted in a private clinic setting that is not fully integrated with broader electronic health record networks, limiting the availability of information on diagnoses and medications obtained outside of NOVI Health.

There was also substantial missingness across several clinical and engagement variables, reflecting real-world implementation constraints. Likewise, adverse events were monitored and addressed as part of routine physician-led clinical care during the program; and no severe adverse events were reported in clinical care (electronic medical record) documentations or at monthly multidisciplinary team meetings that occur as part of NOVI’s standard care model. However, the study dataset did not include structured, quantitative adverse event reporting, and future research would benefit from more formalized capture of such data points alongside clinical outcomes. Additionally, because the program was delivered as an integrated model as a real-world study without a separate control group for the app, medication, or coaching components, we cannot isolate the incremental effect of the digital coaching component, which would be better evaluated through a more traditional randomized control trial design. While app usage metrics served as useful proxies for engagement, they may not fully capture the quality or intent of user interactions. In addition, the dataset lacked information on several potentially important confounders, such as education level, income, and broader social or behavioral factors, which are not routinely collected in clinical care. These limitations should be considered when interpreting the findings, but do not diminish the study’s contribution in generating real-world evidence on the delivery and outcomes of integrated pharmacological and digital obesity care in routine practice.

## Conclusion

In conclusion, this study provides evidence that integrating pharmacological treatment with digitally supported care can lead to meaningful improvements in obesity-related outcomes within a structured program. Beyond the clinical benefits, patterns of participant engagement point to opportunities for more tailored and responsive models of care. As GLP-1 RAs become more widespread for the treatment of diabetes and obesity, embedding behavioral and digital support may be critical to maximizing their long-term impact. For clinical practice, these findings underscore the relevance of multidisciplinary approaches that align with patients’ evolving needs and care environments. Policy efforts should focus on enabling broader and more equitable access to both medication and digital infrastructure. Future research should explore barriers and facilitators to engagement, delineate the conditions that support sustained participation, and deconstruct the influence of Asia-specific social and cultural factors in shaping how individuals experience and respond to these emerging models of obesity care.

## Supplementary information

Supplemental Files

## Data Availability

The datasets and analytic code generated and/or used during the current study are available from the corresponding author on reasonable request.
