# Supplementary material for: Real-world outcomes of hybrid obesity care using digital coaching and GLP-1 therapy in a multi-ethnic Asian setting
Source: Int J Obes (Lond). 2026 Apr 9;50(6):1276–82. doi: 10.1038/s41366-026-02062-x (PMC13286992; doi:10.1038/s41366-026-02062-x)
Supplement: Supplementary file 1 — Supplemental Files [file 41366_2026_2062_MOESM1_ESM.docx]

**Supplemental File 1: Screenshots of the NOVI Health mobile app**


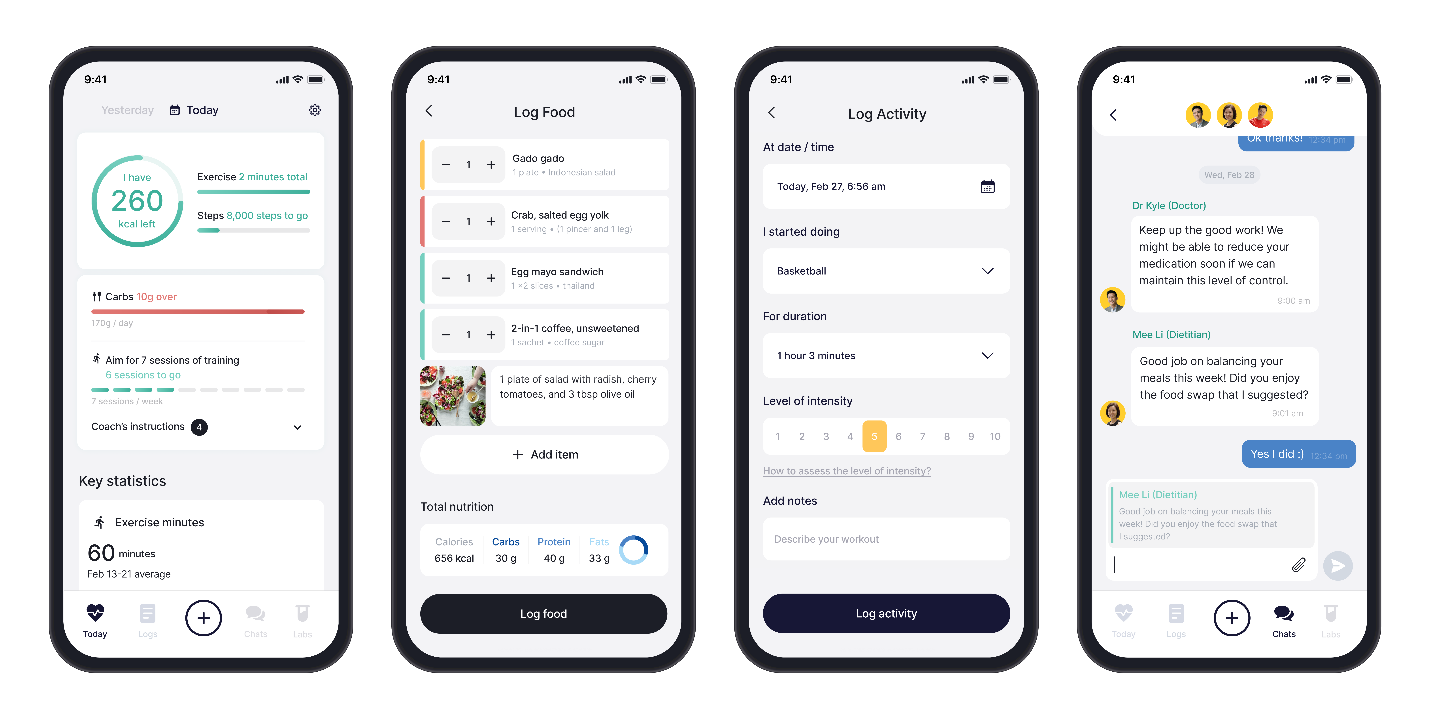


Screenshots of the mobile app dashboard with diet and physical activity goals, dietary intake and physical activity logs, and personalized recommendations delivered through the messaging function.

**Supplemental File 2: Adjusted^1^ odds of greater engagement in NOVI Optimum Plus program across socio-demographic and health characteristics**

| **Variable** | **Program length**  **(>9 months)** | | | **Monthly doctor consults**  **(above median)** | | | **Monthly health coach consults (above median)** | | |
| --- | --- | --- | --- | --- | --- | --- | --- | --- | --- |
| **Age** | **AOR** | **CI** | **p-value** | **AOR** | **CI** | **p-value** | **AOR** | **CI** | **p-value** |
| 21-30 |  |  |  |  |  |  |  |  |  |
| 30-39 | 1.03 | (0.54, 1.98) | 0.932 | 0.63 | (0.34, 1.17) | 0.139 | 0.86 | (0.45, 1.61) | 0.638 |
| 40-49 | 0.99 | (0.52, 1.90) | 0.973 | 0.64 | (0.34, 1.18) | 0.149 | 0.68 | (0.36, 1.27) | 0.236 |
| 50-59 | 1.61 | (0.80, 3.28) | 0.184 | 0.53 | (0.27, 1.04) | 0.066 | 0.55 | (0.27, 1.10) | 0.093 |
| 60+ | 1.58 | (0.59, 4.28) | 0.362 | 0.60 | (0.22, 1.56) | 0.296 | 0.64 | (0.24, 1.68) | 0.362 |
| **Sex** |  |  |  |  |  |  |  |  |  |
| Female |  |  |  |  |  |  |  |  |  |
| Male | 1.08 | (0.77, 1.50) | 0.661 | 0.85 | (0.61, 1.18) | 0.338 | 0.61 | (0.44, 0.85) | 0.003 |
| **Nationality** |  |  |  |  |  |  |  |  |  |
| Non-Singaporean |  |  |  |  |  |  |  |  |  |
| Singaporean | 0.65 | (0.42, 1.00) | 0.049 | 0.96 | (0.64, 1.46) | 0.852 | 1.24 | (0.82, 1.88) | 0.305 |
| **Ethnicity (region)** |  |  |  |  |  |  |  |  |  |
| East Asian |  |  |  |  |  |  |  |  |  |
| South Asian | 0.56 | (0.33, 0.94) | 0.032 | 1.39 | (0.85, 2.27) | 0.190 | 1.34 | (0.82, 2.21) | 0.247 |
| Southeast Asian | 0.38 | (0.20, 0.70) | 0.002 | 1.67 | (0.97, 2.86) | 0.063 | 2.24 | (1.27, 4.04) | 0.006 |
| European | 0.93 | (0.55, 1.55) | 0.769 | 1.44 | (0.87, 2.41) | 0.159 | 1.04 | (0.63, 1.74) | 0.872 |
| Other/Mixed | 0.96 | (0.41, 2.22) | 0.921 | 1.31 | (0.55, 3.02) | 0.526 | 1.11 | (0.48, 2.59) | 0.803 |
| **Health Conditions** |  |  |  |  |  |  |  |  |  |
| Obesity | 1.34 | (0.79, 2.32) | 0.277 | 1.15 | (0.68, 2.00) | 0.609 | 0.97 | (0.57, 1.63) | 0.905 |
| Dyslipidemia | 0.56 | (0.37, 0.82) | 0.004 | 1.22 | (0.84, 1.77) | 0.296 | 1.69 | (1.16, 2.48) | 0.007 |
| Hypertension | 0.63 | (0.40, 0.98) | 0.045 | 1.05 | (0.69, 1.60) | 0.808 | 1.62 | (1.06, 2.49) | 0.028 |
| Hyperglycemia | 2.16 | (1.32, 3.56) | 0.002 | 1.15 | (0.71, 1.84) | 0.565 | 0.50 | (0.31, 0.82) | 0.006 |
| Liver disease | 1.03 | (0.54, 1.98) | 0.932 | 0.63 | (0.34, 1.17) | 0.139 | 0.86 | (0.45, 1.61) | 0.638 |
| **Variable** | **Monthly total logs**  **(above median)** | | | **Monthly app time**  **(above median)** | | | **Monthly messages**  **(above median)** | | |
| **Age** | **AOR** | **CI** | **p-value** | **AOR** | **CI** | **p-value** | **AOR** | **CI** | **p-value** |
| 21-30 |  |  |  |  |  |  |  |  |  |
| 30-39 | 0.58 | (0.29, 1.14) | 0.116 | 1.12 | (0.57, 2.19) | 0.749 | 1.12 | (0.58, 2.17) | 0.743 |
| 40-49 | 0.83 | (0.41, 1.64) | 0.598 | 1.53 | (0.77, 3.02) | 0.222 | 1.59 | (0.82, 3.11) | 0.173 |
| 50-59 | 0.65 | (0.30, 1.38) | 0.266 | 1.31 | (0.61, 2.81) | 0.482 | 1.38 | (0.66, 2.91) | 0.393 |
| 60+ | 0.52 | (0.18, 1.46) | 0.215 | 0.79 | (0.27, 2.25) | 0.663 | 0.84 | (0.30, 2.36) | 0.747 |
| **Sex** |  |  |  |  |  |  |  |  |  |
| Female |  |  |  |  |  |  |  |  |  |
| Male | 0.81 | (0.56, 1.17) | 0.263 | 0.68 | (0.47, 0.99) | 0.047 | 0.84 | (0.58, 1.22) | 0.367 |
| **Nationality** |  |  |  |  |  |  |  |  |  |
| Non-Singaporean |  |  |  |  |  |  |  |  |  |
| Singaporean | 2.07 | (1.32, 3.29) | 0.002 | 2.17 | (1.37, 3.47) | 0.001 | 1.40 | (0.90, 2.20) | 0.136 |
| **Ethnicity (region)** |  |  |  |  |  |  |  |  |  |
| East Asian |  |  |  |  |  |  |  |  |  |
| South Asian | 1.24 | (0.71, 2.17) | 0.455 | 1.23 | (0.70, 2.17) | 0.467 | 1.59 | (0.92, 2.78) | 0.101 |
| Southeast Asian | 1.67 | (0.91, 3.10) | 0.101 | 2.25 | (1.21, 4.30) | 0.012 | 1.51 | (0.84, 2.78) | 0.175 |
| European | 1.71 | (0.97, 3.04) | 0.068 | 1.76 | (0.99, 3.15) | 0.056 | 1.04 | (0.59, 1.82) | 0.893 |
| Other/Mixed | 2.46 | (1.00, 6.31) | 0.054 | 2.33 | (0.94, 6.04) | 0.072 | 1.47 | (0.61, 3.62) | 0.398 |
| **Health Conditions** |  |  |  |  |  |  |  |  |  |
| Obesity | 1.40 | (0.80, 2.50) | 0.240 | 1.33 | (0.76, 2.36) | 0.321 | 1.28 | (0.73, 2.24) | 0.387 |
| Dyslipidemia | 1.69 | (1.12, 2.56) | 0.013 | 1.72 | (1.13, 2.64) | 0.011 | 1.43 | (0.95, 2.15) | 0.088 |
| Hypertension | 1.37 | (0.85, 2.21) | 0.194 | 1.41 | (0.87, 2.30) | 0.165 | 0.79 | (0.50, 1.26) | 0.319 |
| Hyperglycemia | 0.82 | (0.49, 1.39) | 0.463 | 0.57 | (0.33, 0.96) | 0.035 | 0.90 | (0.54, 1.50) | 0.682 |
| Liver disease | 0.58 | (0.29, 1.14) | 0.116 | 1.12 | (0.57, 2.19) | 0.749 | 1.12 | (0.58, 2.17) | 0.743 |
| ^1^ Adjusting for all other variables described above | | | |  |  |  |  |  |  |

**Supplemental File 3: Adjusted^1^ associations between engagement indicators and differential logarithmic monthly changes in health outcomes during the program**

|  | **Change in Weight %** | | | **Change in BMI** | | |
| --- | --- | --- | --- | --- | --- | --- |
|  | Estimate | CI | p-value | Estimate | CI | p-value |
| **Overall estimate** | **-5.05^3^** | **(-5.29, -4.81)** | **<0.001** | **-1.65^3^** | **(-1.74, -1.57)** | **<0.001** |
| **Interaction estimates** |  |  |  |  |  |  |
| Monthly total logs |  |  |  |  |  |  |
| 1st quartile | Ref | | | Ref | | |
| 2nd quartile | -0.72 | (-1.08, -0.35) | <0.001 | -0.11 | (-0.24, 0.03) | 0.120 |
| 3rd quartile | -0.97 | (-1.33, -0.60) | <0.001 | -0.35 | (-0.48, -0.21) | <0.001 |
| 4th quartile | -2.00 | (-2.37, -1.63) | <0.001 | -0.65 | (-0.79, -0.52) | <0.001 |
| Monthly messages |  |  |  |  |  |  |
| 1st quartile | Ref | | | Ref | | |
| 2nd quartile | -0.74 | (-1.11, -0.37) | <0.001 | -0.31 | (-0.45, -0.18) | <0.001 |
| 3rd quartile | -1.55 | (-1.93, -1.17) | <0.001 | -0.49 | (-0.63, -0.35) | <0.001 |
| 4th quartile | -1.02 | (-1.39, -0.64) | <0.001 | -0.32 | (-0.46, -0.18) | <0.001 |
| Monthly app time |  |  |  |  |  |  |
| 1st quartile | Ref | | | Ref | | |
| 2nd quartile | -1.11 | (-1.46, -0.76) | <0.001 | -0.33 | (-0.46, -0.20) | <0.001 |
| 3rd quartile | -1.56 | (-1.92, -1.19) | <0.001 | -0.56 | (-0.70, -0.43) | <0.001 |
| 4th quartile | -2.15 | (-2.51, -1.78) | <0.001 | -0.72 | (-0.85, -0.58) | <0.001 |
| Monthly doctor consults |  |  |  |  |  |  |
| 1st quartile | Ref | | | Ref | | |
| 2nd quartile | 0.17 | (-0.16, 0.49) | 0.316 | 0.02 | (-0.09, 0.14) | 0.680 |
| 3rd quartile | 0.49 | (0.17, 0.82) | 0.003 | 0.17 | (0.06, 0.29) | 0.004 |
| 4th quartile | 0.87 | (0.42, 1.32) | <0.001 | 0.29 | (0.12, 0.45) | <0.001 |
| Monthly health coach consults^2^ |  |  |  |  |  |  |
| Low (Below median) | Ref | | | Ref | | |
| High (Above median) | -0.36 | (-0.61, -0.12) | 0.003 | -0.13 | (-0.22, -0.05) | 0.0027 |
|  | **Change in WHR** | | | **Change in SBP^4^** | | |
|  | Estimate | CI | p-value | Estimate | CI | p-value |
| **Overall estimate** | **-0.0103** | **(-0.0127, -0.008)** | **<0.001** | **-4.62** | **(-5.59, -3.66)** | **<0.001** |
| **Interaction estimates** |  |  |  |  |  |  |
| Monthly total logs |  |  |  |  |  |  |
| 1st quartile | Ref | | | Ref | | |
| 2nd quartile | 0.0000 | (-0.0036, 0.0036) | 0.994 | -2.67 | (-4.68, -0.66) | 0.009 |
| 3rd quartile | -0.0004 | (-0.0039, 0.00003) | 0.802 | -1.30 | (-3.32, 0.72) | 0.206 |
| 4th quartile | -0.0021 | (-0.0055, 0.0013) | 0.235 | -2.91 | (-4.95, -0.87) | 0.005 |
| Monthly messages |  |  |  |  |  |  |
| 1st quartile | Ref | | | Ref | | |
| 2nd quartile | -0.0051 | (-0.0085, -0.0016) | 0.004 | -1.82 | (-3.67, 0.02) | 0.053 |
| 3rd quartile | -0.0066 | (-0.0103, -0.0030) | <0.001 | -1.85 | (-3.83, 0.14) | 0.068 |
| 4th quartile | -0.0024 | (-0.0057, 0.0009) | 0.151 | -0.6 | (-2.56, 1.36) | 0.548 |
| Monthly app time |  |  |  |  |  |  |
| 1st quartile | Ref | | | Ref | | |
| 2nd quartile | 0.0024 | (-0.0010, 0.0058) | 0.161 | -2.45 | (-4.72, -0.18) | 0.035 |
| 3rd quartile | -0.0029 | (-0.0063, 0.0005) | 0.099 | -2.16 | (-4.22, -0.10) | 0.040 |
| 4th quartile | -0.0014 | (-0.0048, 0.0020) | 0.420 | -2.38 | (-4.53, -0.24) | 0.030 |
| Monthly doctor consults |  |  |  |  |  |  |
| 1st quartile | Ref | | | Ref | | |
| 2nd quartile | -0.0034 | (-0.0067, -0.0001) | 0.044 | -0.26 | (-2.78, 2.26) | 0.840 |
| 3rd quartile | -0.0021 | (-0.0054, 0.0011) | 0.199 | -0.91 | (-3.36, 1.54) | 0.464 |
| 4th quartile | 0.0017 | (-0.0029, 0.0063) | 0.468 | 1.24 | (-2.15, 4.64) | 0.472 |
| Monthly health coach consults^2^ |  |  |  |  |  |  |
| Low (Below median) | Ref | | | Ref | | |
| High (Above median) | -0.0017 | (-0.0041, 0.0007) | 0.160 | 1.14 | (-0.23, 2.50) | 0.103 |
|  | **Change in Fat %** | | | **Change in Total Chol** | | |
|  | Estimate | CI | p-value | Estimate | CI | p-value |
| **Overall estimate** | **-3.53** | **(-4.90, -2.16)** | **<0.001** | **-4.08** | **(-12.13, 3.96)** | **0.319** |
| **Interaction estimates** |  |  |  |  |  |  |
| Monthly total logs |  |  |  |  |  |  |
| 1st quartile | Ref | | | Ref | | |
| 2nd quartile | -2.67 | (-4.68, -0.66) | 0.009 | -2.71 | (-13.47, 8.05) | 0.621 |
| 3rd quartile | -1.30 | (-3.32, 0.72) | 0.206 | -14.02 | (-24.88, -3.16) | 0.012 |
| 4th quartile | -2.91 | (-4.95, -0.87) | 0.005 | -5.95 | (-16.28, 4.39) | 0.258 |
| Monthly messages |  |  |  |  |  |  |
| 1st quartile | Ref | | | Ref | | |
| 2nd quartile | -1.82 | (-3.67, 0.02) | 0.053 | 3.97 | (-7.14, 15.07) | 0.483 |
| 3rd quartile | -1.85 | (-3.83, 0.14) | 0.068 | -3.36 | (-14.97, 8.25) | 0.569 |
| 4th quartile | -0.60 | (-2.56, 1.36) | 0.548 | -5.56 | (-15.15, 4.04) | 0.255 |
| Monthly app time |  |  |  |  |  |  |
| 1st quartile | Ref | | | Ref | | |
| 2nd quartile | -2.45 | (-4.72, -0.18) | 0.035 | -1.29 | (-11.92, 9.34) | 0.811 |
| 3rd quartile | -2.16 | (-4.22, -0.10) | 0.040 | -6.19 | (-17.10, 4.71) | 0.265 |
| 4th quartile | -2.38 | (-4.53, -0.24) | 0.030 | -3.08 | (-14.11, 7.96) | 0.584 |
| Monthly doctor consults |  |  |  |  |  |  |
| 1st quartile | Ref | | | Ref | | |
| 2nd quartile | -0.26 | (-2.78, 2.26) | 0.840 | -2.72 | (-13.48, 8.03) | 0.619 |
| 3rd quartile | -0.91 | (-3.36, 1.54) | 0.464 | -6.07 | (-17.12, 4.98) | 0.281 |
| 4th quartile | 1.24 | (-2.15, 4.64) | 0.472 | -4.06 | (-16.69, 8.57) | 0.527 |
| Monthly health coach consults^2^ |  |  |  |  |  |  |
| Low (Below median) | Ref | | | Ref | | |
| High (Above median) | 1.14 | (-0.23, 2.50) | 0.103 | -7.24 | (-14.54, 0.05) | 0.052 |
|  | **Change in HDL Chol** | | | **Change in LDL Chol** | | |
|  | Estimate | CI | p-value | Estimate | CI | p-value |
| **Overall estimate** | 0.36 | (-1.55, 2.27) | 0.710 | -3.23 | (-10.11, 3.65) | 0.146 |
| **Interaction estimates** |  |  |  |  |  |  |
| Monthly total logs |  |  |  |  |  |  |
| 1st quartile | Ref | | | Ref | | |
| 2nd quartile | 2.75 | (0.27, 5.23) | 0.030 | -3.64 | (-13.04, 5.77) | 0.448 |
| 3rd quartile | 2.38 | (0.01, 4.75) | 0.049 | -11.53 | (-20.70, -2.37) | 0.014 |
| 4th quartile | 2.78 | (0.43, 5.14) | 0.021 | -9.62 | (-18.66, -0.59) | 0.037 |
| Monthly messages |  |  |  |  |  |  |
| 1st quartile | Ref | | | Ref | | |
| 2nd quartile | 2.43 | (-0.07, 4.93) | 0.056 | 2.31 | (-7.31, 11.93) | 0.637 |
| 3rd quartile | 2.81 | (0.39, 5.22) | 0.023 | -7.32 | (-16.74, 2.11) | 0.128 |
| 4th quartile | 3.59 | (1.52, 5.66) | <0.001 | -8.80 | (-17.00, -0.61) | 0.035 |
| Monthly app time |  |  |  |  |  |  |
| 1st quartile | Ref | | | Ref | | |
| 2nd quartile | 2.56 | (0.29, 4.83) | 0.027 | -4.7 | (-13.42, 4.03) | 0.290 |
| 3rd quartile | 2.21 | (-0.21, 4.62) | 0.073 | -8.42 | (-17.71, 0.88) | 0.076 |
| 4th quartile | 2.44 | (0.06, 4.82) | 0.044 | -6.97 | (-16.27, 2.34) | 0.142 |
| Monthly doctor consults |  |  |  |  |  |  |
| 1st quartile | Ref | | | Ref | | |
| 2nd quartile | 2.79 | (0.44, 5.15) | 0.020 | -5.91 | (-14.65, 2.84) | 0.185 |
| 3rd quartile | 3.70 | (1.26, 6.13) | 0.003 | -8.10 | (-17.14, 0.94) | 0.079 |
| 4th quartile | 1.11 | (-1.57, 3.78) | 0.416 | -3.84 | (-13.85, 6.17) | 0.451 |
| Monthly health coach consults^2^ |  |  |  |  |  |  |
| Low (Below median) | Ref | | | Ref | | |
| High (Above median) | 1.41 | (-0.26, 3.09) | 0.098 | -7.96 | (-14.15, -1.78) | 0.012 |
|  | **Triglycerides** | | | **Change in HbA1c** | | |
|  | Estimate | CI | p-value | Estimate | CI | p-value |
| **Overall estimate** | -8.68 | (-21.77, 3.71) | 0.169 | **-0.22** | **(-0.30, -0.14)** | **<0.001** |
| **Interaction estimates** |  |  |  |  |  |  |
| Monthly total logs |  |  |  |  |  |  |
| 1st quartile | Ref | | | Ref | | |
| 2nd quartile | -18.54 | (-34.33, -2.75) | 0.022 | 0.14 | (0.02, 0.25) | 0.018 |
| 3rd quartile | -13.07 | (-28.15, 2.00) | 0.089 | 0.08 | (-0.03, 0.18) | 0.137 |
| 4th quartile | -15.05 | (-29.96, -0.13) | 0.048 | 0.14 | (0.03, 0.25) | 0.010 |
| Monthly messages |  |  |  |  |  |  |
| 1st quartile | Ref | | | Ref | | |
| 2nd quartile | -1.28 | (-17.28, 14.71) | 0.875 | -0.06 | (-0.18, 0.06) | 0.307 |
| 3rd quartile | -6.03 | (-21.63, 9.58) | 0.448 | -0.02 | (-0.13, 0.09) | 0.757 |
| 4th quartile | -11.71 | (-25.34, 1.93) | 0.092 | 0.09 | (-0.01, 0.2) | 0.074 |
| Monthly app time |  |  |  |  |  |  |
| 1st quartile | Ref | | | Ref | | |
| 2nd quartile | -2.19 | (-16.67, 12.28) | 0.766 | -0.04 | (-0.14, 0.06) | 0.441 |
| 3rd quartile | -14.47 | (-29.88, 0.94) | 0.066 | 0.03 | (-0.09, 0.14) | 0.645 |
| 4th quartile | -8.49 | (-23.88, 6.90) | 0.278 | 0.05 | (-0.07, 0.17) | 0.438 |
| Monthly doctor consults |  |  |  |  |  |  |
| 1st quartile | Ref | | | Ref | | |
| 2nd quartile | -5.57 | (-21.14, 10.00) | 0.482 | -0.15 | (-0.25, -0.06) | 0.002 |
| 3rd quartile | -11.50 | (-27.60, 4.59) | 0.161 | 0.05 | (-0.06, 0.16) | 0.388 |
| 4th quartile | -9.42 | (-27.71, 8.86) | 0.312 | 0.06 | (-0.06, 0.18) | 0.300 |
| Monthly health coach consults^2^ |  |  |  |  |  |  |
| Low (Below median) | Ref | | | Ref | | |
| High (Above median) | -10.14 | (-21.23, 0.94) | 0.073 | 0.01 | (-0.06, 0.09) | 0.727 |

WHR: waist-to-hip ratio; SBP: systolic blood pressure; ^1^ Adjusting for age, sex, nationality, ethnicity, and total health conditions, and interaction effects of sex and total health conditions. ^2^ Due to the distribution of health coach engagement data, a median split was used instead of quartiles. ^3^ Sensitivity analyses restricted to in-clinic measurements (46.8% of weight change and 52.1% of BMI datapoints analyzed) showed minimal differences from the main analysis: weight change –4.95 (95% CI: –5.28, –4.61); BMI change –1.69 (95% CI: –1.82, –1.57). ^4^ Only SBP findings are displayed, as DBP findings were similar.
